# Tests and Studies of the Ocular Muscles

**Published:** 1899-06

**Authors:** 


**Tests and Studies of the Ocular Muscles.** By ERNEST E. MADDOX, M.D. Pp. xv., 427. Bristol: John Wright and Co. 1898.

Those who are familiar with the work of Dr. Maddox on ophthalmological prisms will anticipate in this volume a useful and interesting book; and they will not be disappointed. The problems dealt with are admittedly difficult and complex, but the author's clearness and precision render them comparatively simple; his happy and well-chosen expressions, his ingenious methods and luminous suggestions, convert a somewhat dry and tedious subject into a most interesting study.

We know of no book where so good a description of Tenon's capsule is given; but what will probably be found the most useful portions of the book are the chapters on ocular paralysis. These are so admirably clear, so carefully led up to, and so copiously illustrated, that if they are consulted a mistake in diagnosis is scarcely possible. Numerous clever contrivances and ingenious applications of already existing methods and apparatus are described. The author's "torsion calculator," which anyone can construct for himself, illustrates very prettily how, in certain cases of ocular paralysis, torsion of the false image is brought about; and new uses for the "tangent scale" are explained. The difficult and hitherto much neglected subject of "latent torsion" of the eyeball deserves special mention as indicating an advance in ophthalmology, and an attempt to discover a cause and possibly a remedy for certain cases of eye-strain.

Every chapter, every page almost, bears the stamp of the author's originality, and the diagrams and general get-up of the book leave nothing to be desired.

